# Machine-learning-based prediction of the effectiveness of the delivered dose by exhale-gated radiotherapy for locally advanced lung cancer: The additional value of geometric over dosimetric parameters alone

**DOI:** 10.3389/fonc.2022.870432

**Published:** 2023-01-13

**Authors:** Nika Guberina, Christoph Pöttgen, Alina Santiago, Sabine Levegrün, Sima Qamhiyeh, Toke Printz Ringbaek, Maja Guberina, Wolfgang Lübcke, Frank Indenkämpen, Martin Stuschke

**Affiliations:** ^1^ Department of Radiation Therapy, West German Cancer Center, University Hospital Essen, University Duisburg-Essen, Essen, Germany; ^2^ German Cancer Consortium (DKTK), Partner Site University Hospital Essen, Essen, Germany

**Keywords:** Hausdorff-distance, cold spot, respiratory gating, planning target volume margin, lung cancer, clinical target volume

## Abstract

**Purpose:**

This study aimed to assess interfraction stability of the delivered dose distribution by exhale-gated volumetric modulated arc therapy (VMAT) or intensity-modulated arc therapy (IMAT) for lung cancer and to determine dominant prognostic dosimetric and geometric factors.

**Methods:**

Clinical target volume (CTV_Plan_) from the planning CT was deformed to the exhale-gated daily CBCT scans to determine CTV_i_, treated by the respective dose fraction. The equivalent uniform dose of the CTV_i_ was determined by the power law (*g*EUD_i_) and cell survival model (EUD_iSF_) as effectiveness measure for the delivered dose distribution. The following prognostic factors were analyzed: (I) minimum dose within the CTV_i_ (D_min_i_), (II) Hausdorff distance (HDD_i_) between CTV_i_ and CTV_Plan_, (III) doses and deformations at the point in CTV_Plan_ at which the global minimum dose over all fractions per patient occurs (PD_min_global_i_), and (IV) deformations at the point over all CTV_i_ margins per patient with the largest Hausdorff distance (HDPw_orst_). Prognostic value and generalizability of the prognostic factors were examined using cross-validated random forest or multilayer perceptron neural network (MLP) classifiers. Dose accumulation was performed using back deformation of the dose distribution from CTV_i_ to CTV_Plan_.

**Results:**

Altogether, 218 dose fractions (10 patients) were evaluated. There was a significant interpatient heterogeneity between the distributions of the normalized *g*EUD_i_ values (*p*<0.0001, Kruskal–Wallis tests). Accumulated *g*EUD over all fractions per patient was 1.004–1.023 times of the prescribed dose. Accumulation led to tolerance of ~20% of fractions with *g*EUD_i_ *<*93% of the prescribed dose. Normalized D_min_ >60% was associated with predicted *g*EUD values above 95%. D_min_ had the highest importance for predicting the *g*EUD over all analyzed prognostic parameters by out-of-bag loss reduction using the random forest procedure. Cross-validated random forest classifier based on D_min_ as the sole input had the largest Pearson correlation coefficient (R=0.897) in comparison to classifiers using additional input variables. The neural network performed better than the random forest classifier, and the *g*EUD values predicted by the MLP classifier with D_min_ as the sole input were correlated with the *g*EUD values characterized by R=0.933 (95% CI, 0.913–0.948). The performance of the full MLP model with all geometric input parameters was slightly better (R=0.952) than that based on D_min_ (*p*=0.0034, Z-test).

**Conclusion:**

Accumulated dose distributions over the treatment series were robust against interfraction CTV deformations using exhale gating and online image guidance. D_min_ was the most important parameter for *g*EUD prediction for a single fraction. All other parameters did not lead to a markedly improved generalizable prediction. Dosimetric information, especially location and value of D_min_ within the CTV**_i_**, are vital information for image-guided radiation treatment.

## Introduction

Gated radiotherapy in plain free breathing delivered during the exhalation phase is efficient with duty cycles of 30%–50% of the breathing cycle. In comparison to ungated irradiation, gated radiotherapy can substantially reduce the residual tumor motion during irradiation and consequently the doses to organs at risk (1–3). By nature, the major tumor movement is observed in lesions located adjacent to the heart, aorta, or diaphragm (4). Gated cone beam CTs (CBCT) at the end of expiration can further enhance the precision of lung tumor localization by reducing motion artifacts (5). In the past, image-guided radiotherapy based on CBCTs was primarily performed by matching implanted markers (6), a rigid 3–6-degrees-of-freedom carina or bony match (7, 8) or a rigid soft-tissue match. The latter may be achieved automatically or by aligning the center of mass and orientation of the small peripheral targets (9–12). Motion at distinct points, e.g., implanted markers or center of masses of target volumes, were traditionally used for PTV margin calculation under simplifying assumptions (13). The real-time position management (RPM) respiratory gating system allows reproducible tumor gating as external marker (14). In addition, gated radiotherapy can reduce interfractional setup uncertainties (15). However, for larger target volumes, considerable interfractional displacements (>5 mm) may be observed between the primary tumor and draining lymph nodes, characterized by systematic and random errors (10, 16). Furthermore, anatomical changes owing to tumor shrinkage can occur throughout the course of curative-intended radiation therapy. These make adaptations of the target volume necessary in order to protect normal tissue (17–21).

The purpose of this analysis is the characterization of the dosimetric and geometric parameters related to the residual deformations of the clinical target volume (CTV) in prefractional, exhale-gated CBCT scans after 6-degrees-of-freedom image guidance. Furthermore, the influence of the residual deformations on the delivered equivalent uniform dose (EUD) at each dose fraction shall be examined. Fast identification of relevant features related to the effectiveness of the dose fraction during online image guidance of radiotherapy is of high importance in order to minimize avoidable deviations of the delivered from the prescribed EUD to the target volume. The EUD represents the homogeneously delivered dose distribution within the target that leads to the same clonogenic survival fraction; thus, the same level of cell kill, as the actual non-homogeneously delivered, absorbed dose distribution (22). The EUD is determined according to the phenomenological power law model (*g*EUD) (23) or a cell survival model as effectiveness measure (EUD_SF_) (22). As the final end point, the accumulated dose shall be determined over all fractions per patient to assess the impact of residual deformations on the overall effectiveness of the treatment series and to find evidence for a possible reduction of clinical PTV margins below 5 mm. The prognostic performance of geometric features characterizing the residual deformations of the CTV and dosimetric characteristics at distinct points of the CTV shall be analyzed to predict the *g*EUD and EUD_SF_ per dose fraction.

## Materials and methods

### Patient characteristics

The study was approved by the local ethics committee (20-9293-BO). Patients with histologically confirmed non-small or small-cell lung cancer who underwent either definitive or neoadjuvant radiochemotherapy after interdisciplinary tumor board consensus and radiotherapeutic indication were included into this dosimetric study. Depending on the optimal plan solution, patients were treated with a hyper-fractionated [1.5 Gy bid (twice a day)] or with a conventionally fractionated [(2 Gy qed (once a day)] therapy regimen in free breathing gated delivery during the exhalation phase according to clinical standards.

### Imaging and treatment planning

Helical, contrast-enhanced computed tomography (CT), positron emission tomography/computed tomography ([^18^F]FDG-PET/CT), EBUS-TBNA (ultrasound-controlled transbronchial needle aspiration), and clinical assessment (***viz.***, laboratory parameters, ECOG performance status, and lung function parameters) were completed prior to treatment planning. After visual and acoustic breathing coaching, a prospective gated planning CT in expiration was acquired with contrast medium (gating threshold, below one-third of the inspiratory amplitude). The planning CT was acquired in supine position in exhalation and as a 4D-CT at a multislice CT scanner (Siemens Healthineers, Erlangen, Forchheim, Germany). Minimization of motion may be achieved by a rigid half-body mask (in the present study, 9/10 patients). Treatment planning was performed with the help of the treatment planning system Eclipse (version V15.5, Varian Medical Systems, Palo Alto, USA). The target volume definition was according to RTOG0617 (24). The IASLC lung cancer map (25) and the Japanese Society for Radiation Oncology atlas were used for the description of involved lymph node stations (26). For patients receiving induction chemotherapy, gross tumor volume (GTV) was delineated on the basis of the post-induction chemotherapy planning CT and on the initial pre-treatment contrast-enhanced imaging and the pretreatment PET/CT in order to account for infiltrated sites and initially involve critical structures such as bronchi and intrapulmonal vessels. CTV was contoured with 5–10 mm margins around the GTV not crossing anatomic boundaries. To consider setup errors, the PTV margin was set to 5 mm, which, in individual cases, could be adapted according to the daily CBCTs. Patients who underwent a definitive radiochemotherapy received a total dose of 60–66 Gy delivered once daily (2 Gy/F qed) with or without subsequent boost. In the neoadjuvant radiochemotherapy setting prior to thoracic surgery, a total dose of 45 Gy twice daily was administered. Treatment plans were optimized by qualified medical physicists with ACUROS XB dose calculation algorithm (implemented in treatment planning system Eclipse version 15.5.) using 6–8 MV photons and a 2×2×2 mm voxel grid. Dose prescription was in accordance with International Commission on Radiation Units and Measurement ICRU 83. PTV coverage was set to D80% ≥ 100% and D95% ≥ 98%. Thresholds for organs at risk and normal tissue dose–volume constraints for conventionally fractionated radiotherapy with concurrent chemotherapy were specified according the NCCN guideline, Version 1.2023 (27).

### Radiotherapy treatment delivery and daily image guidance

Treatment was delivered at a TrueBeam linac (Varian Medical Systems, Palo Alto, USA) equipped with a 6-degrees-of-freedom couch. Cone-beam computed tomography (CBCT) can be acquired by a gantry mounted X-ray line. All patients were treated with 6 or 8 MV photons using static field IMRT or volumetric modulated arc therapy technique (RapidArc, Varian Medical Systems, Palo Alto, USA), which enables favorable gradients around the target volume (28).

Free breathing gated delivery during the exhalation phase was provided with the help of real-time position management (RPM) respiratory gating system (Varian). Daily low-dose kV-CBCT scans also gated during the exhalation phase served for the online setup and online adaptation with the help of a 6D-positioning table at TrueBeam (Varian) and were used for the determination of interfractional CTV deformations (CTV_i_) compared to the planning CT (CTV_plan_). Further tools used for online image guidance were set up with anterior/posterior kV radiographs, breathing coaching to a flat stable respiration at the expiration plateau, and anterior/posterior fluoroscopy with delineation of the diaphragm for documentation of the respiration amplitude relative to the RPM marker.

### CTV deformations

In order to examine the daily deviations from patient anatomy, the planning CT, which served as reference, was deformed offline to the CBCT_i_ scans after alignment by the prefractional, rigid online match clinically applied (Eclipse v15.5, Varian). The matching region of interest was centered around the carina. The deformable registration implemented in Eclipse (Eclipse v15.5, Varian) relies on a modified, accelerated demons-based algorithm (29, 30). The deformed CTV_i_ was copied back to the planning CT along the rigid online registration.

The Hausdorff distance (HDD_i_) between CTV_i_ and CTV_Plan_ was determined by the radius of an isotropic expansion of CTV_Plan_ as the minimum expansion, so that the expanded CTV_plan_ contained CTV_i_. HDP_worst_ is the Hausdorff point (HDP_i_) with the largest HDD_i_ over all fractions per patient. We determined the deformations of the global HDP_worst_i_ over all fractions by determining the position of HDPw_orst_ on CTV_plan_ by the inverse deformation vector to HDP_worst_ and by recording the deformation fields from CTV_plan_ to CTV_i_ at that point over all fractions. In addition to HDP_worst_ the dose fraction with the smallest minimum dose in the CTV_i_ over all dose fractions per patient j was determined, and the coordinates of the respective global minimum point PD_min_global_ per patient j was determined on CTV_plan_ by the inverse deformation vector to this point. D_min_global_ is the global minimum overall fractions of the minimum doses in the deformed CTV_i_ for each fraction (D_min_CTVi_); propagating PD_min_global_ to all CTV_i_s by the respective deformation fields defined the coordinates of the PD_min_global_i_ points on the CTV_i_ and allowed the recording of the doses at PD_min_global_i_. Furthermore, we determined for each fraction i the scalar product of the deformation vector from PD_min_global_ to PD_min_global_i_ and the unit deformation vector from PD_min_global_ to PD_min_global_x_ with x as the fraction in which the global minimum dose was observed (Scalar_PDmin_global_i_x_) (**Supplementary Figure S1**, new). This feature is a surrogate for the component of deformation to PD_min_global_i_ in the direction of the steepest dose gradient.

In general, dose–volume histograms for the CTV_i_ were obtained from the dose distribution calculated on CT_plan_. According to the static dose cloud approximation, the dose distribution might not be influenced by the small deformations between CT_plan_ and CBCT_i_ (31). To check this assumption, the dose distribution was recalculated on the planning CT deformed by the deformation matrix to CBCT_i_. The ARIA deformable registrations were exported to MIM Maestro software (MIM Software Inc., Cleveland, OH) to re-raster the CT data set into parallel layers for dose calculation. Dose was calculated with the Acuros XB algorithm on this re-rastered deformed planning CT. Gamma analysis of two given dose distributions was performed in MIM.

### Equivalent uniform dose

In order to estimate the overall effectiveness of the delivered dose distribution to the CTV_i_, the original equivalent uniform dose (EUD) based on the clonogen survival model (EUD_SF_) (22) and the phenomenological power law model as (*g*EUD) (23, 32) was applied to the dose–volume histogram data for the CTVi (tissue-specific parameter a= −20 for tumor). For the calculation of the EUD_SF_, the alpha/beta value was determined as 4 Gy according to Nix et al. (33). The cell number per tumor was regarded as 100,000,000, and a uniform cell density was assumed (34). SF_2_ resulting in tumor control rates of 50% at 60 Gy with 2 Gy per fraction was calculated as 0.534598229. For the calculation of the *g*EUD and EUD_SF_ values, dose–volume histograms (DVHs) for the deformed CTV_i_ were written out of the Eclipse system and calculated in SAS (SAS Institute, Cary, NC).

### Dose accumulation

A central component for dose accumulation is deformable image registration (DIR). The AAPM Task Group 132 has produced guidelines for quality control of DIR algorithms, which state that results should be qualitatively assessed by image fusion of the deformed image and the reference, with emphasis on matching anatomical landmarks and boundaries (35). Two image deformation algorithms were used here, namely, Varian’s SmartAdapt V 13.6, based on an accelerated demon algorithm (36), and hybrid intensity- and structure-based algorithms from MIM Software, Cleveland, OH (37). In this study, all deformed images and structures were analyzed by an experienced thoracic radiologist and radiation oncologist. In particular, CTV boundaries were analyzed in relation to anatomical landmarks, such as mediastinal landmarks or the pericardium. In case of important violations of these boundaries, the margins of the CTV_i_ were manually corrected in order to respect this relation. In all cases in which the supervised CTV_i_ was manually corrected, the deformation vector field was updated using the hybrid structure-based deformation algorithm in MIM. Dose accumulation was performed with the help of the MIM software. The dose cubes on the planning CT were deformed with the inverse deformation matrix from the planning CT to the CBCT_i_ obtained from the clinical Eclipse planning software for the manually uncorrected CTV_i_ or by the deformation matrix of the supervised corrected CTV_i_ to the CTV by the hybrid intensity and structure based algorithm from MIM software. These deformed dose cubes for each fraction were summed up to the cumulative dose. This cumulative dose cube was exported to Eclipse, and the DVH for the original CTV_plan_ was determined (**Supplementary Figure S2** new).

### Statistical analysis and machine learning models

For statistical analyses, SAS software version 9.4, SAS/STAT 15.1 (SAS Institute, Cary, NC) and SPSS Statistics (version 26, IBM, Armonk, NY) were applied. *p*-values were considered two-sided, and *p*<0.05 was considered as statistically significant. Various SAS procedures including FREQ, UNIVARIATE, NPAR1WAY, CORR, GLM, HPNEURAL, and HPFOREST were used.

The high-performance analytical procedures HPFOREST and HPNEURAL of SAS Enterprise Miner 14.3 were used to create the random forest (RF) and multilayer perceptron neural network (MLP) models (SAS Institute, Cary, NC) (SAS Institute Inc. 2020, SAS® Interprise Miner 15.2™:: High performance Procedures, SAS Institute Inc., Cary, NC). The sensitivity of MLP classifier was calibrated using the relative weight factor of 2 for *g*EUD_i_ values between 90% and 95% (relative weight=2) and 10 for the identification of *g*EUD_i_ values <90%. The relative weights of all other *g*EUD_i_ values were 1.

To assess the generalization performance of the classifier, 10-fold cross-validation was used, leaving out each patient and scoring the data from this patient by the classifier trained on the data from the other patients. This was repeated 10 times for all leave-out patients. As HPNEURAL uses a validation data set to tune hyperparameters, nested cross-validation was used (38). The outer resampling trainings data sets contained data from nine patients and were subdivided into an inner loop trainings data set containing data from six patients and the inner loop validation data set containing data of the remaining three patients. The inner loop classifier with the best correlation between the predicted *g*EUD_i_ and the true *g*EUD_i_ values obtained directly from the dose–volume histograms for the nine patients in the outer resampling trainings set was used to score the leave-out patient. This leave-out patient in the test data set was unseen during classifier build.

The strength of a linear relation between the true *g*EUD_i_ values calculated directly from the dose–volume histograms for the CTV_i_ from the different fractions and the *g*EUD_i_ values predicted by a classifier was assessed by the Pearson correlation coefficient. Correlation coefficients from either two MLP or random forest classifiers with different input variable sets were compared by a Z-test for dependent correlation coefficients (39). In addition, the variance explained by the predictive model based on cross-validation (VE_cv_) was examined (40). The performance of the classifiers by the VE_cv_ measure using D_min_ alone or the complete feature set as input was compared using the signed rank test.

## Results

Altogether, 218 fractions from 10 consecutive patients were included in this study (median number of CBCT_i_ per patient, 12.00; mean, 13.82; range, 11–40; SD, 9.52). Mean patient age was 68.8 years (range, 54–83 years). Exhale-gated daily low-dose kV CBCT scans were performed for online image guidance using a 6-degrees-of-freedom table. All patients had histopathologically confirmed lung cancer (nine NSCLC and one SCLC) and underwent neoadjuvant (n=1) or definitive (n=9) radiochemotherapy treatment after interdisciplinary tumor board consensus. Mean FEV1 in lung function test at initial staging was 2.0 l (61.3%/set point). The clinical characteristics, tumor features, localization, and clinical target volumes of these patients are given in **Table 1**. After expert radiologists and radio-oncologists supervision, deformed CTV_i_ boundaries were found as incorrect in 14/218 fractions, crossing afore-respected boundaries and therefore were manually corrected. All supervised and as reliable determined deformations were used for further analyses.

**Table 1:** Tumor characteristics and radiotherapy technique.

Patient	Tumor entity	Lobe	Side	CTV volume (cm³)	Respiratory gating window	Technique	Number of examined fractions with CBCT	Treatment strategy
**A**	NSCLC	Lower	Left	149.8	15-80%	VMAT	28	Neoadjuvant
**B**	NSCLC	Upper	Left	176.1	30-80%	VMAT	25	Definitive
**C**	NSCLC	Lower	Left	151.5	10-80%	VMAT	33	Definitive
**D**	SCLC	Lower	Right	321.0	10-77%	Static IMRT	11	Definitive
**E**	NSCLC	Lower	Right	344.2	30-70%	Static IMRT	12	Definitive
**F**	NSCLC	Lower	Right	177.8	20-80%	VMAT	40	Definitive
**G**	NSCLC	Lower	Light	170.0	25-80%	Static IMRT	30	Definitive
**H**	NSCLC	Upper	Left	228.2	25-80%	Static IMRT	13	Definitive
**I**	NSCLC	Lower	Right	231.5	25-80%	Static IMRT	14	Definitive
**J**	NSCLC	Upper	Left	149.3	10-80%	VMAT	12	Definitive

Depiction of tumor characteristics (entity; location; clinical target volume (cm³)), and the applied radiotherapy technique (static field IMRT or volumetric modulated arc therapy technique (RapidArc, Varian Medical Systems, Palo Alto, USA)), number of examined fractions with CBCT, and treatment strategy (neoadjuvant or definitive radiochemotherapy).

**Figure 1:**
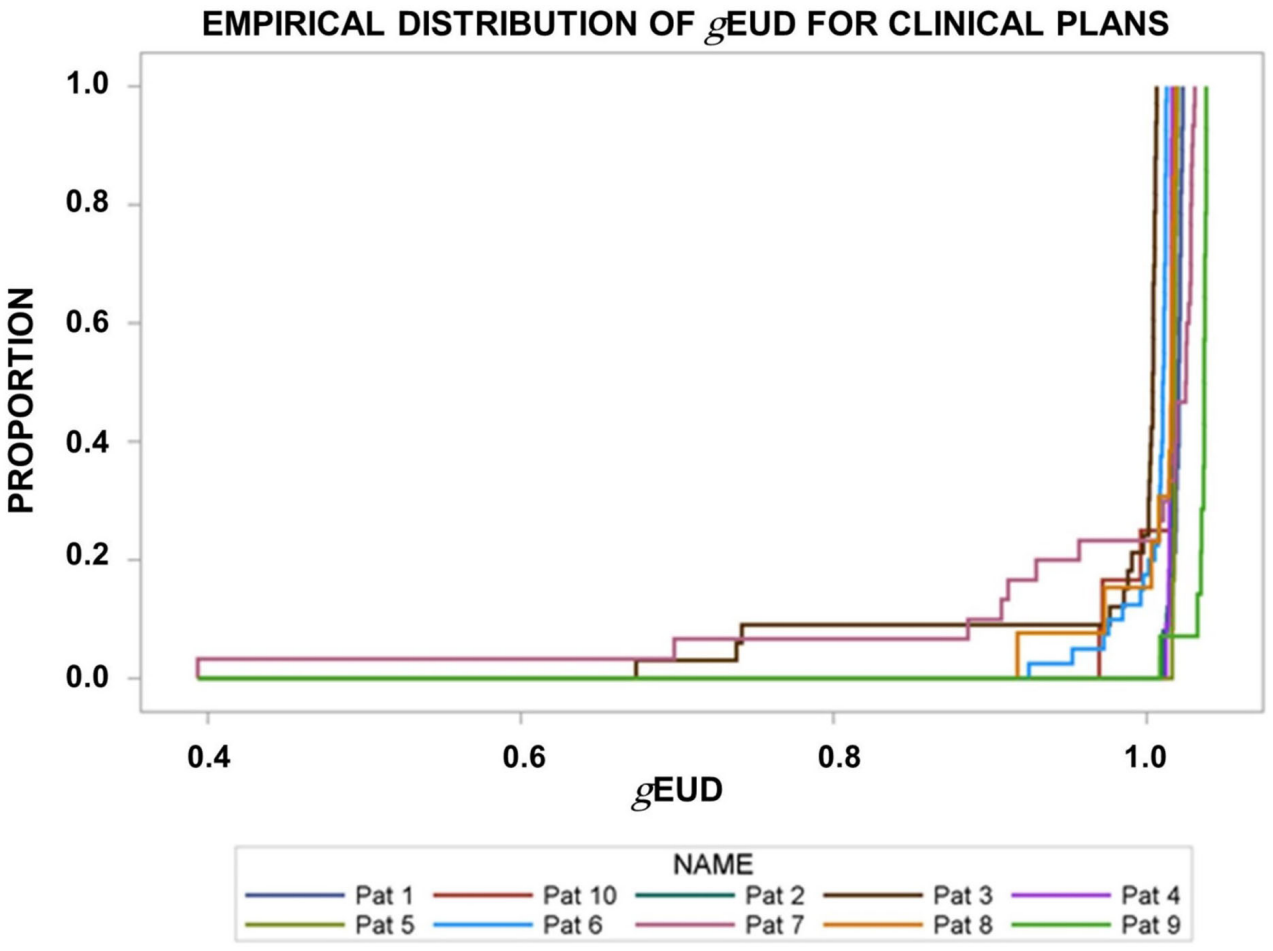
Empirical distribution functions of the generalized equivalent uniform dose (*g*EUDi) for the deformed CTV_i_ on the pre-fraction cone beam CTs normalized by the prescribed dose. The clinically applied dose distributions were used. There was significant interpatient heterogeneity (*p*< 0.0001, Kruskal–Wallis test).

**Figure 2:**
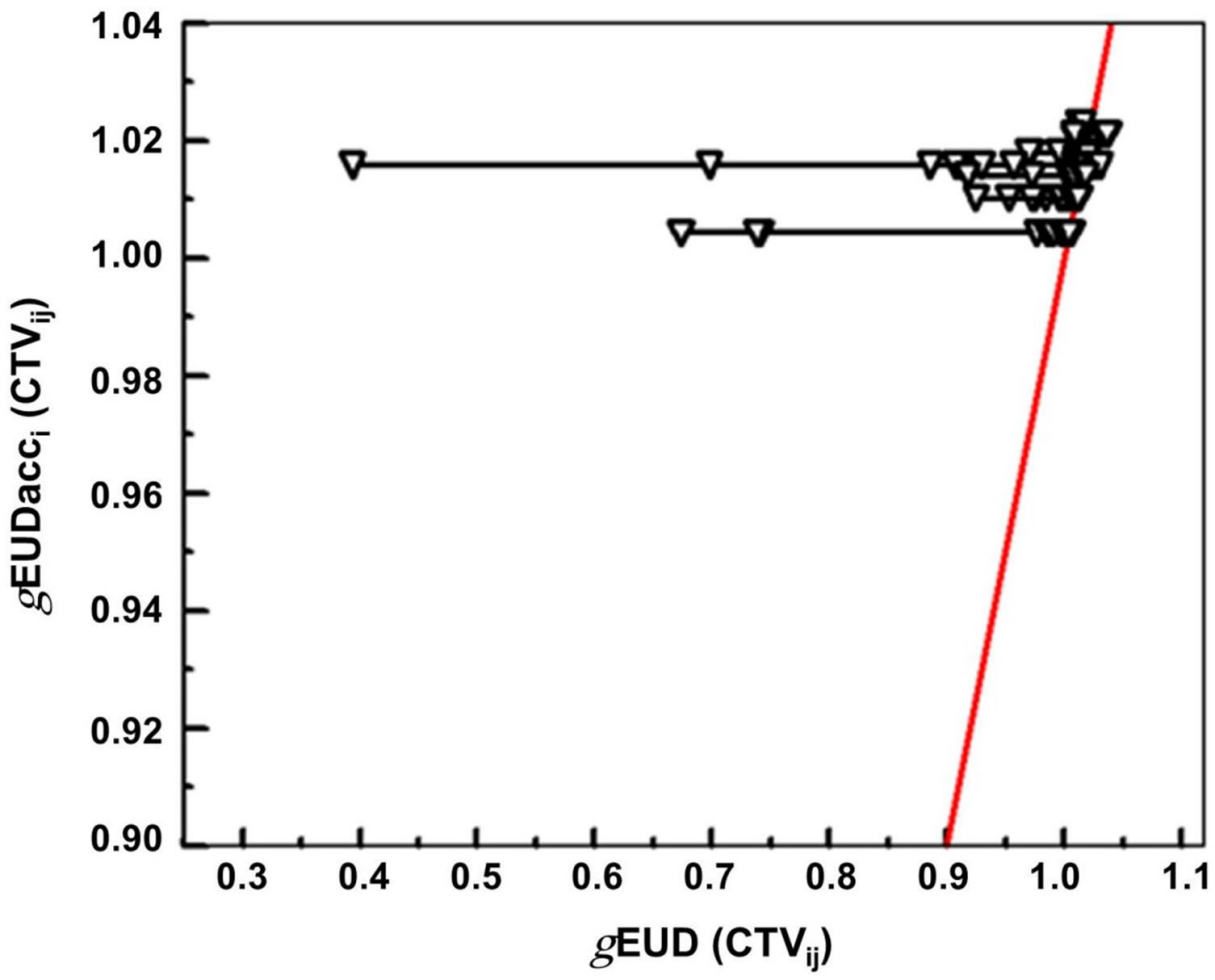
Plot of the normalized *g*EUD_i_ values for the different dose fractions of a series versus the normalized *g*EUD for the accumulated dose distribution. The data are given for the clinical plan for the 10 patients. The red line represents the 1:1 line connecting the accumulated *g*EUD_acc_j_ values for the different patients.

**Figure 1** shows the empirical distribution functions of the *g*EUD_i_ values for the deformed CTV_i_ over the radiotherapy series for the different patients using the clinically applied dose distribution optimized on planning target volumes around the CTV_Plan_ with a 5-mm margin. There was a significant interpatient heterogeneity between the *g*EUD_i_-distribution functions from the different patients (*p* < 0.0001, Kruskal–Wallis tests). All *g*EUD_i_s were normalized to the prescribed dose. The median *g*EUD_i_ over all fractions per patient fell in the narrow range between 1.004 and 1.037 for the different patients (**Figures 1**, **2**). *g*EUD_i_ for 11 of the 218 fractions from four patients fell below 93% of the prescribed dose. The dose gradients around the PTV were steeper around the superior and inferior surface of the PTV than at the equatorial surface at z-coordinates around the geometric center of the PTV. The median dose gradients between the 95% and the 70% isodose in cranial direction around the cranial and caudal direction at the caudal PTV border were 8.34%/mm (range, 5%/mm–12.5%/mm). The median normalized dose gradient in axial direction at z-coordinates around the geometric center of the PTV was 3.12%/mm (range, 1.92–6.25%/mm) toward the mediastinum and 2.5%/mm (range, 1.47–6.25%/mm) toward the lateral thorax.

The static dose cloud approximation was analyzed in five fractions from three patients. The pass rate inside of the region of interest, i.e., CTV+15 mm, remained for the five examined fractions above 95% for the 3 mm distance to agreement (DTA) and 3% dose difference (DD) gamma criteria. The pass rates for the stricter 2 mm DTA, 2% DD criterion remained above 89%. The *g*EUD values calculated for the CTV_i_ using the recalculated dose distributions on the deformed planning CTs deviated from those calculated from the original dose distribution from the planning CT by a median value of 1.6%.

**Figure 3:**
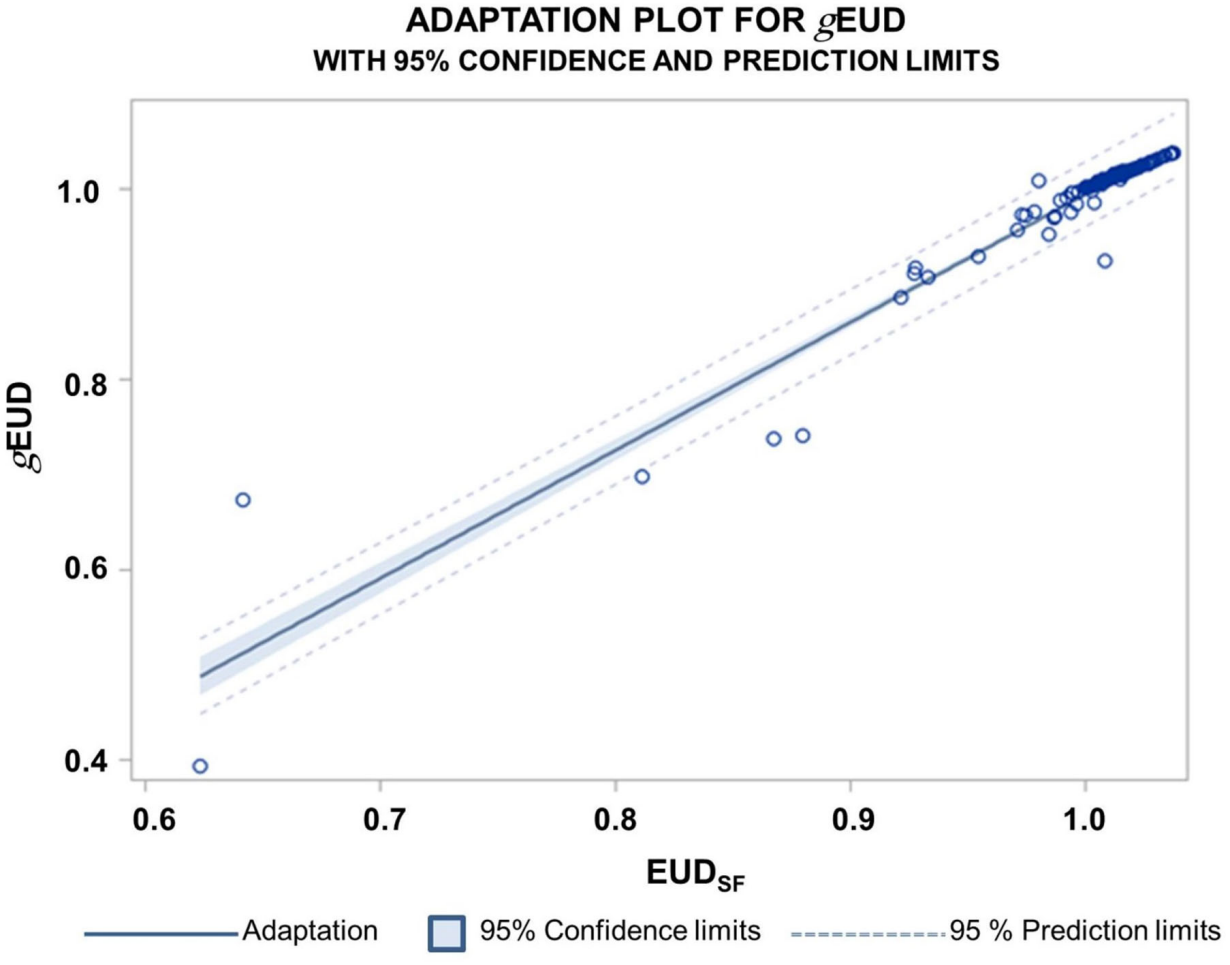
Relation between the *g*EUD normalized by the prescribed dose and normalized EUD_SF_ values for the clinical PTV plans over all patients and all fractions. The normalized EUD_SF_ values were calculated according to a cell survival model. The Spearman correlation coefficient was r_s_ = 0.986; 95% confidence limits for the expected predicted values and for new predictions are indicated.

For all patients, the *g*EUD of the accumulated dose distribution over all fractions (*g*EUD_acc_) remained above 100% of the prescription dose for the CTV_Plan_. Dose accumulation helped to raise the *g*EUD_acc_ over all fractions per patient near the median of the *g*EUD_i_ distribution overall fractions per patient (**Table 2**). **Figure 2** shows the scatter plot for *g*EUD_i_ (CTV_ij_) versus the *g*EUD_acci_ values. The red line is the 1:1 bisector line. This plot shows how tolerant the *g*EUD_acc_ behaves against dose deviations at a single fraction leading to declines in *g*EUD_i_. *g*EUD_acc_ for the accumulated dose distribution was stable against observed minimum doses of the accumulated dose distributions down to 72% of the prescribed dose. There was a good correlation between the *g*EUD_i_ and EUD_SFi_ values over all fractions and patients (Spearman rank correlation coefficient r_s_ = 0.986, *p* < 0.0001). The best relation comprised an intercept of −0.35 ± 0.027 and a slope of 1.346 ± 0.027 (**Figure 3**).

**Table 2:** Delivered dose parameters by the clinical plans and measures for the residual CTV deformations after image guidance.

Patient	*g*EUD of the accumulated dose distribution	D_min_ of the accumulated dose distribution	Median *g*EUD_i_	Min *g*EUD_i_	Min EUD_SFi_
**A**	1.021	81.7	1.020	1.008	1.006
**B**	1.017	88.4	1.016	1.010	1.008
**C**	1.004	95.1	1.004	0.674	0.641
**D**	1.023	86.1	1.015	1.012	1.011
**E**	1.017	88.3	1.018	1.016	1.016
**F**	1.010	80,1	1.010	0.925	0.974
**G**	1.016	72.4	1.025	0.394	0.623
**H**	1.014	81.1	1.016	0.917	0.928
**I**	1.021	86.1	1.037	1.009	1.009
**J**	1.019	81.7	1.017	0.970	0.987

Depiction of gEUD of the accumulated dose distribution, D_min_ of the accumulated dose distribution, median and min gEUD_i_, for the different fractions i of the treatment series, min EUD_SFi_, for the clinical plans. All EUDs are normalized to the prescribed doses. EUD_SF_ was calculated for a treatment series of 30 fractions at 2 Gy per fraction, repeatedly applying the dose distribution of the considered fraction.

**Figure 4:**
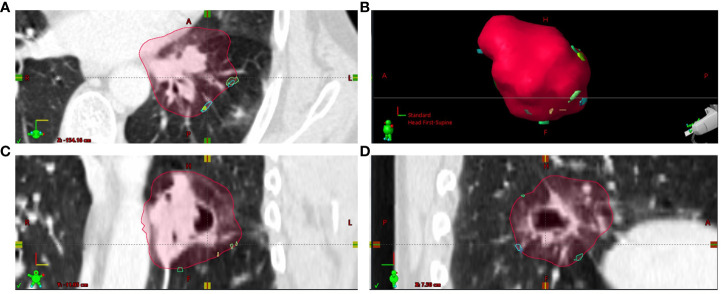
The localizations of Hausdorff points from the different dose fractions i (HDP_i_) transferred to CT_plan_ by deformable image registration are shown at the outer edge of the CTV_plan_ contour for patient **(A)** Most HDP_i_ on the CTV_plan_ contour were inferior to the CTV_plan_ center. **(A)** Axial plane through CTV_plan_; **(B)** 3D view on CTV_plan_ surface; **(C)** frontal plane through CTV_plan_; **(D)** sagittal plane through CTV_plan_.

**Figure 5:**
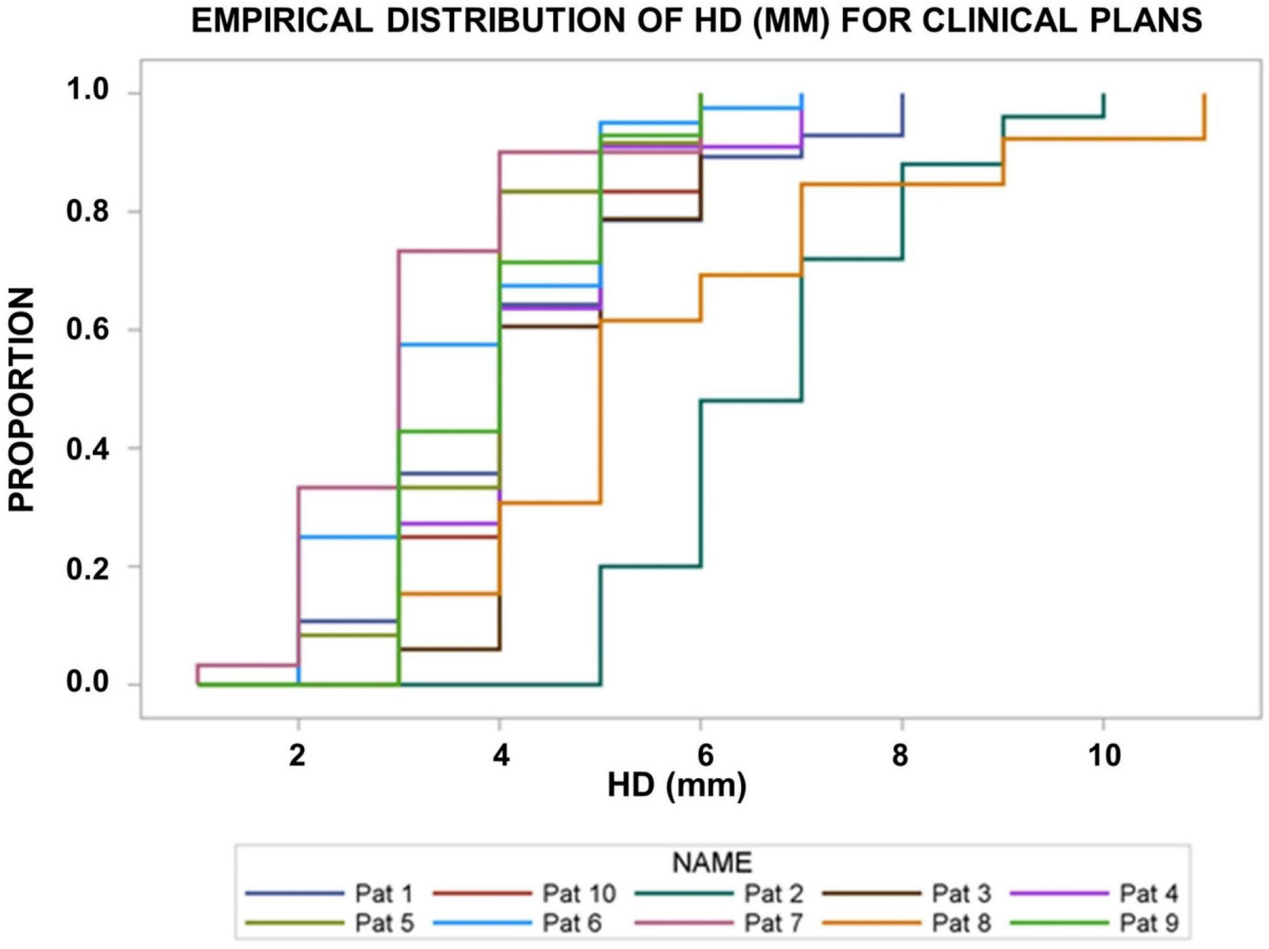
Empirical distribution functions of the Hausdorff distances (HD, in mm) of the deformed CTV_i_ on the pre-fraction cone beam CTs’ from the original CTV in the planning CT. There was significant interpatient heterogeneity (*p*< 0.0001, Kruskal–Wallis test).

With respect to parameters characterizing the residual deformations of the CTV_i_s, the Hausdorff distances (HDD_i_) as the maximum over the minimum distances between the deformed and original CTV borders were determined (**Figure 4** new). **Figure 5** depicts the empirical distribution functions (EDFs) for the HDD_i_ values for different patients with significant differences from patient to patient (*p* < 0.0001, Kruskal–Wallis test). The median HDD_i_ values for the different patients ranged from 3.0 to 7.0 mm.

**Figure 6:**
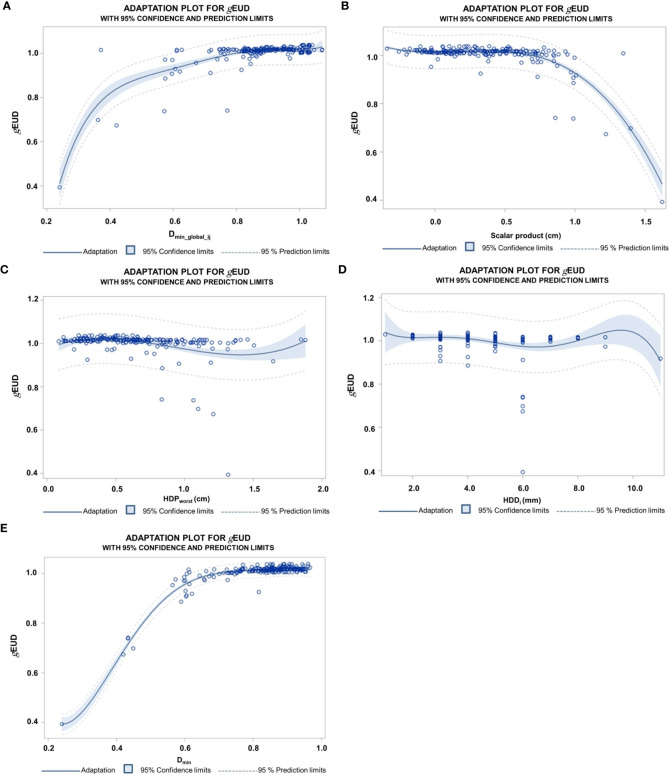
**(A)** Dependence of *g*EUD values normalized by the prescribed dose on the normalized dose at the global D_min_ point (D_min_global_ij_) over all 218 fractions from the 10 patients approximated by a polynomial fit. The highest significant degree of the polynomial terms was 5; the coefficient of determination R square was 0.69. **(B)** Polynomial fit of the normalized *g*EUD_i_ by the length of the deformation vector at the global minimum point of the CTV overall fractions per patients in the direction of the steepest dose decline (Scalar_PDmin_global_i_x_). The normalized *g*EUD_i_ remained stable up to deformations of 8 mm, followed by a steep descent. The highest degree of a significant term was 3; the coefficient of determination was 0.69. The clinical PTV margin was 5–6 mm. **(C)** Polynomial regression of the normalized *g*EUD_i_ values on the length of deformation vectors at HDP_worst_ (ΔL-HDP_worst_). HDP_worst_ represents the point on the CTV margin per patient with the largest Hausdorff distance to the deformed CTV_i_ overall fractions and was given in cm. The degree of the polynomial is 3; the coefficient of determination is 0.13. **(D)** Fit of the normalized *g*EUD values by the Hausdorff distances HDD_i_ between the deformed CTV_i_ per fraction and the CTV on the planning CT overall fractions from the different patients. The degree of the polynomial is 5; the coefficient of determination is 0.08. **(E)** Fit of the *g*EUDi values normalized by the prescribed dose in dependence on the normalized D_min_ over the 218 fractions from 10 patients. The degree of the fitted polynomial is 5; the coefficient of determination is 0.94.

In **Figures 6A–E**, we analyzed the relations between geometric and dosimetric deviations at two distinct points, namely, the PD_min_global_, the point with the lowest dose in the deformed CTV_i_s over all fractions per patient, the HDP_worst_, the Hausdorff point with the largest distance over all fractions, and the *g*EUD_i_ per fraction. **Figure 6A** shows the dependence of the *g*EUD_i_ values on the normalized dose at PD_min_global_ over all fractions from all patients (D__PDmin_global_i_). The regression curve was fitted up to the highest significant degree of D__PDmin_global_i_ that was 3. The coefficient of determination (R**^2^**) as goodness-of-fit measure was 0.694. The F-value for the fit with 3 degrees of freedom was F=96.2 (*p* < 0.0001). A similar goodness of fit was observed for a fit of *g*EUD_i_ by Scalar_PDmin_global_i_x_ (**Figure 6B**). The highest significant degree of freedom was again 3, R**^2^** for the fit was 0.687, and the F-value for the fit was 156.3 with 3 degrees of freedom. Markedly smaller R**^2^** were found for the fits of *g*EUD_i_ by the geometric Hausdorff parameters. **Figure 6C** shows the relation of *g*EUD_i_ and the length of the deformation vector at the global Hausdorff point (ΔLi-HDP_worst_). The highest significant degree of freedom of the polynomial was 3, R**^2^** was 0.126, and the F-value for the model was 10.2 with 3 degrees of freedom (*p* < 0.0001). **Figure 6D** shows the fit of *g*EUD_i_ by the HDD_i_ values, with the highest significant degree of 3. The goodness of fit measure was even lower with R**^2^** of 0.08 and an F-value for the model of 3.7 with 3 degrees of freedom (*p* = 0.0031). A much closer relation was found between *g*EUD_i_ and the minimum dose in the CTV_i_ per fraction (D_min_CTVi_), characterized by a coefficient of determination of 0.94 and an F-value for the model with 5 degrees of freedom of 680.7 (*p* < 0.0001) (**Figure 6D**). The use of a multivariable model to explain *g*EUD_i_ by the above five parameters up to their highest significant degree from univariable analysis revealed only D_min___CTVi_ (*p* < 0.0001) and HD_worst_ length (*p* < 0.0005) as simultaneously significant.

**Figure 7:**
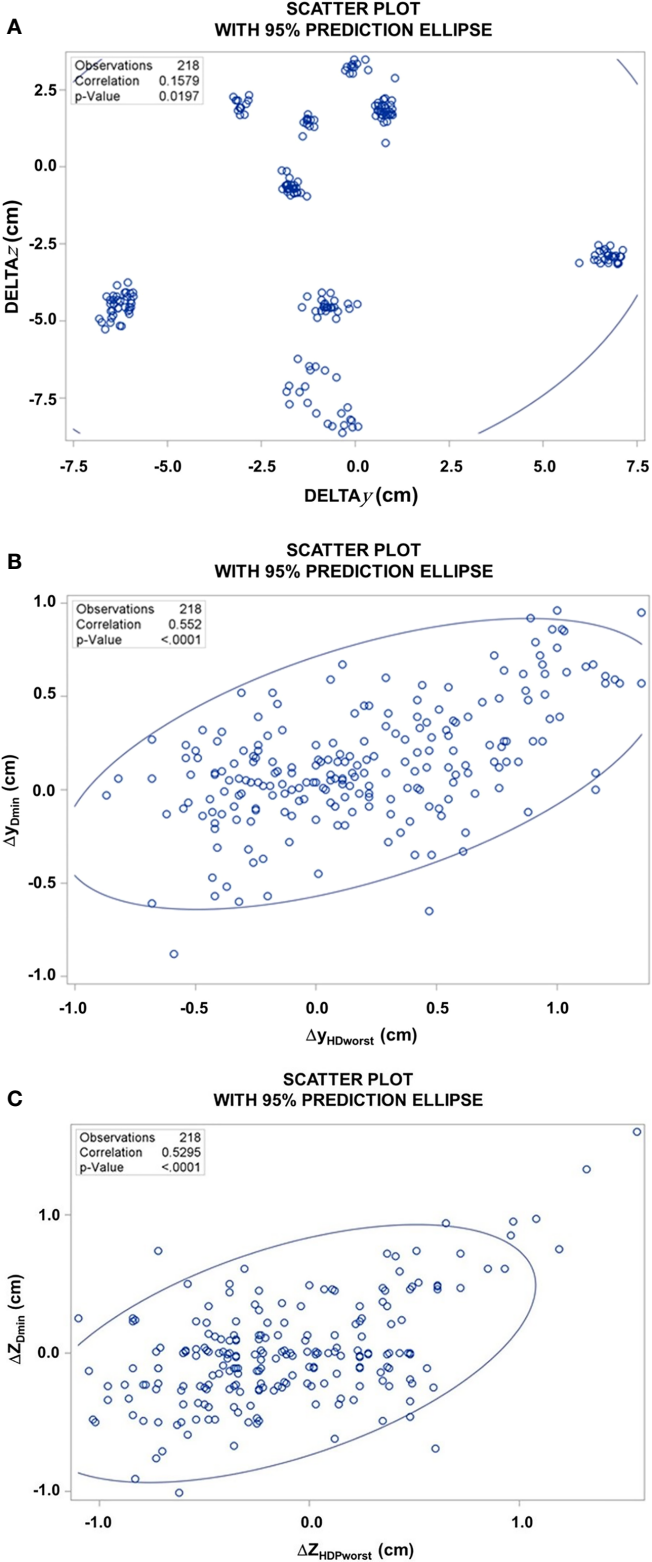
**(A)** Scatter plot of the difference of the *z-*coordinates of PD_min_global_i_ and HDP_worst_i,_ versus the respective difference of the *y*-coordinates over all 218 fractions from the 10 patients, DELTA*z* [Z_HDPWorst_ – Z_PDmin_global_] and DELTA*y* [Y_HDPWorst_ – Y_PDmin_global_]. **(B)** Scatter plot of the y-coordinates of the deformation vectors at of PD_min_global_i_ versus HDP_worst_. **(C)** Scatter plot of the z-coordinates of the deformation vectors at PD_min_global_i_ versus HDP_worst_ over all 218 fractions.

The deformations of the CTV between the two points PD_min_global_i_ and HDP_worst_i_ are depicted in **Figures 7A–C**. Depending on the patient, there was a marked difference between the absolute locations of the points over the fractions for the different patients (**Figure 7A**). The deformations in *y* and *z* directions at these points showed a broad scatter and only a moderate correlation approximately 0.5, demonstrating that there is considerable randomness in the deformations at different points of the CTV (**Figures 7B, C**). The random component of the differences in the deformation vectors in x, y, and z directions were 3.0, 3.3, and 3.9 mm. The systematic differences in the deformation vectors between these points in x, y, and z directions as the standard errors of the distributions of average differences per patient over all patients were 1.7, 3.0, and 2.3 mm, respectively.

**Figure 8:**
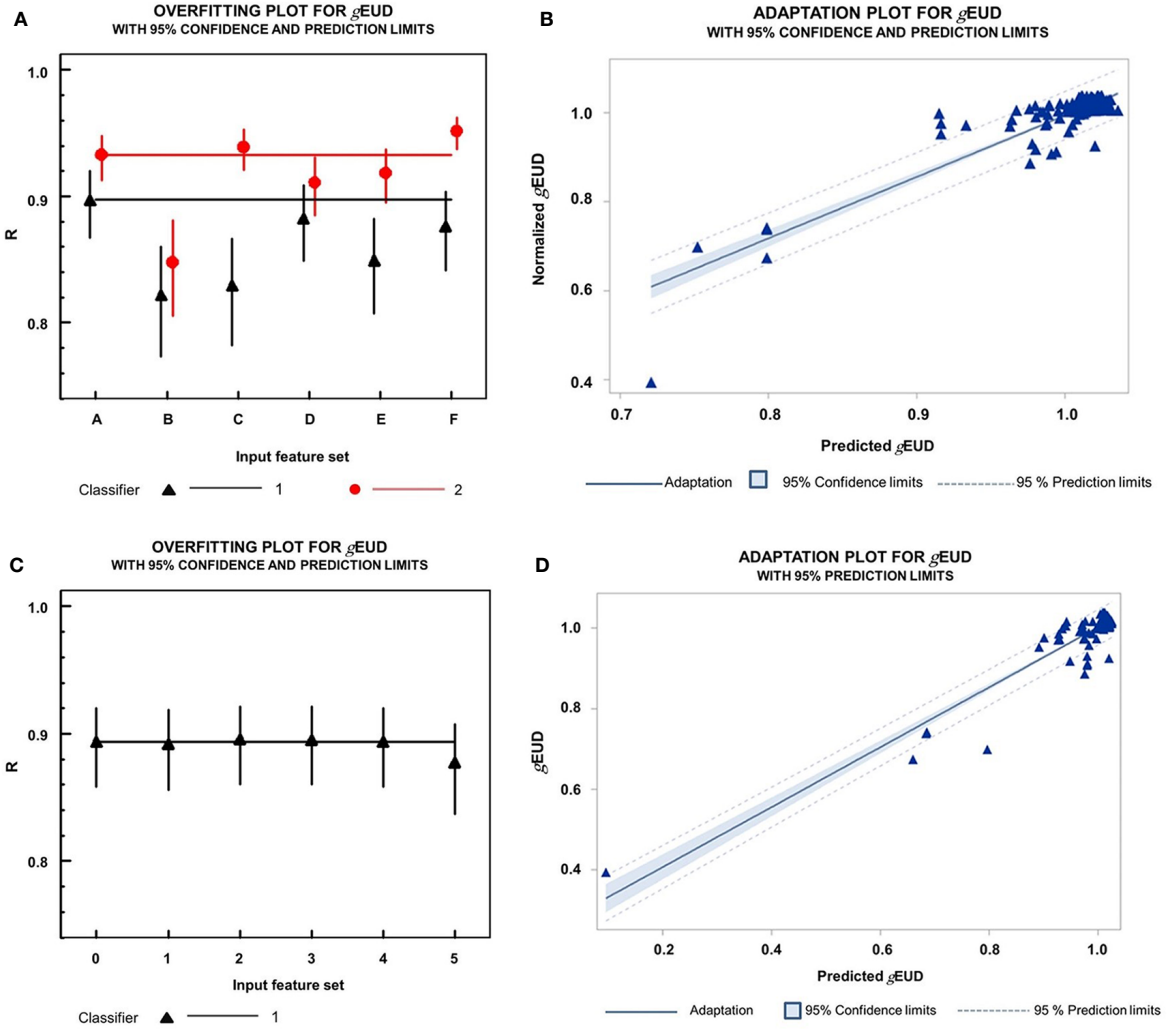
**(A)** Overfitting plot on the dependence of classifier performance with 10-fold cross-validation on the composition of the input feature set. The Pearson correlation coefficients R for the closeness of a linear relation between the *g*EUDi values directly calculated from the dose–volume histograms of the 218 fractions and the *g*EUDi values predicted by the respective classifier. Black triangles up 1 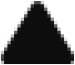: cross-validated random forest classifiers. Red circles 2 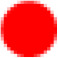: nested cross-validated MLP classifiers. Correlation coefficients are given together with their 95% confidence intervals using Fisher’s transformation. Input feature sets for the different classifiers were as follows: A, D_min_; B, D_min_ and scalar_PDmin_global_i_x_; C, D_min_, scalar_PDmin_global_i_x_, D__PDmin_global_; D, D_min_ and patient ID; E, D_min_, patient ID and HDD; F, D_min_, scalar_PDmin_global_i_x_, D__PDmin_global_, HDDi, ΔL-HDP_worst_, and patient-ID. Classifier: 1: MLP neural net using 90% of the whole data set as a training and 10% as validation data set; 2. MLP with outer loop cross-validation; 80% of the data set was used for training, 10% for validation, and 10% for outer loop validation. Comparison of the correlation coefficients for the random forest classifiers revealed that B, C, and E classifiers all were significantly worse than A at alpha=0.01 (Z-test). The same hold for D and E classifiers in comparison to A for the MLP classifiers at alpha=0.01 (Z-test). Only the F-MLP classifier using the full set of input variables was slightly better than A (*p*=0.003, Z-test). **(B)** Linear relation between the true normalized *g*EUD_i_ values versus the normalized *g*EUD_i_ values predicted by a random forest classifier with D_min_ alone as input feature using 10-fold cross-validation. The Pearson correlation coefficient is 0.897 (95% CI, 0.867–0.920). **(C)** Dependence of cross-validated random forest classifier performance for prediction of *g*EUD_i_ for all fractions after fraction 5 with D_min_ as the sole input variable on the composition of the trainings data set: 0, contained data from all other patients and no data from the leave out patient; 1, contained in addition data from fraction 1 of the leave out patient. i (ε 2–5), contained in addition data from fraction 1 to i of the leave out patient. R, Pearson correlation coefficient between the true *g*EUD_i_ values and the *g*EUD_i_ values predicted by the respective classifier together with their 95% confidence intervals. **(D)** Linear relation between the true normalized *g*EUD_i_ values versus the normalized *g*EUD_i_ values predicted by an MLP neural net classifier with D_min_ alone as input feature using 10-fold nested cross-validation. The Pearson correlation coefficient is 0.933 (95% CI, 0.913–0.948).

In the next step, we evaluated two machine learning model types for the prediction of the *g*EUD_i_ by the above characterized input features, namely, D_min_, D__PDmin_global_, HDD_i_, Scalar_PDmin_global_i_x_, and ΔL-HDP_worst_, and patient ID, indicating the data belonging to the same patient. The performance of classifiers with 10-fold cross-validation was measured by the Pearson correlation coefficient between the true *g*EUD_i_ values from the dose–volume histograms and the *g*EUD values predicted by the classifier. The importance of the different input variables for the random forest classifier was quantified by mean square error loss reduction by the variable using the out-of-bag data (SAS Institute Inc., 2015, SAS® Enterprise miner™ 14.1: High-performance procedures, SAS Institute Inc., Cary, NC). During cross-validation loops, D_min_ always showed the highest importance, followed by Scalar_PDmin_global_i_x_ and D__PDmin_global_. HDD and ΔL-HDP_worst_ did not lead to consistent loss reductions. **Figure 8A** shows an overfitting plot, plotting the performance of the cross-validated random forest classification in dependence on the input features sets from D_min_ alone to the full model containing all the above-mentioned variables. The Pearson correlation coefficient R was 0.897 (95% CI, 0.867–0.920) for the simplest model containing D_min_ alone and was not improved by the more complex models using more features. **Figure 8B** shows the linear relation between the true *g*EUD_i_ and predicted *g*EUD_i_ values by the cross-validated random forest classifier based on D_min_ alone. Furthermore, we analyzed whether the knowledge of the relation between features from fractions 1 to 5 and the respective *g*EUD_i_ values of the considered patient j in addition to the data from the other patients improves the predictions of the *g*EUD_i_ for the following fractions >5 of the scored leave-out patient. **Figure 8C** shows the dependence of the performance of the cross-validated random forest classifier based on D_min_ alone in dependence of the stepwise introduction of data from fraction 1 to 5 of the considered leave-out patient in the trainings data sets. No improvements in the correlation coefficients between the true *g*EUD values and those predicted by the classifier were observed.

In addition, MLP classifiers with nested cross-validation were studied. While their performance was generally better than that of the random forest model, no consistent improvement was observed for models containing more variables than D_min_ alone. The Pearson correlation coefficient between true and predicted *g*EUD_i_ values by the MLP classifier based on D_min_ was R = 0.933 (95%CI, 0.913–0.948) (**Figure 8D**). In addition, we analyzed the performance of the MLP classifier based on the additional geometric input features D__PDmin_global_, HDD_i_, Scalar_PDmin_global_i_x_, and the related ΔL-HDP_worst_, and patient ID using nested cross-validation (**Figure 8A**). The Pearson correlation coefficients for the correlation between true and predicted *g*EUD_i_ was generally slightly better for the MLP in comparison to the random forest classifiers using the same input feature sets. The differences in performance became significant by the VE_cv_ criterion using the input data sets a and c (*p*<0.05, signed rank test), while no significant differences were observed for the other input sets (**Figure 8A**). Comparing the correlation coefficients as a performance measure among the MLP classifiers, only the prediction based on the full input data set was slightly better with R = 0.952 (95% CI, 0.937–0.963) than the predictions based on D_min_ as input alone (*p* = 0.0034, z-test). Both the widely used r-value as a measure for predictive accuracy and the VE_cv_ measure led to the same result, in which the accuracy of the cross-validated random forest and MLP classifiers could not be increased by adding geometrical data in addition to D_min_ into the input feature set.

As D_min_i_ was by far the best single predictor for the *g*EUD_i_, we analyzed the correlation between other parameters from the high-dose region of the dose–volume histograms for the CTVi and D_min_i_ using the 20 fractions with the lowest normalized D_min_i_ from the 218 fractions studied. The correlation with D_min_i_ degraded from D99.9 over D99 and D98 to D50. The respective correlation coefficients were 77.5%, 59.0%, 55.7%, and 1.7%.

## Discussion

The steepness of the dose–control curves for patients with locally advanced lung cancer decreases with intertumor heterogeneity in biological characteristics. This influences radio-responsiveness and impacts dosimetric parameters for dose coverage (34, 41). Retrospective analyses showed the existence of a dose–response relation for locally advanced non-small-cell lung cancer (NSCLC) *(* 42 *)*. In addition, a randomized trial on escalation of the biological effective dose in stage-I NSCLC by stereotactic ablative radiotherapy showed a significant dose–response relation (43). However, an increasing response relation with dose was not found in the large RTOG-0617-trial, which compared standard (60 Gy) versus high-dose (74 Gy) radiation with concurrent chemotherapy for stage-III NSCLC with or without Cetuximab *(* 44 **)**. Exploratory *post-hoc* analyses were performed, which found an association of worse tumor control rates with higher doses to cardio-pulmonary structures *(* 45). Data on the delivered doses to the CTV or data on image guidance were not given. However, the median of the actually applied minimum margin between PTV and CTV was 3.9 mm (range, 0 – 9.8 mm) smaller in the dose-escalated groups than 4.5 mm (range, 0.0–9.8 mm) in the 60 Gy total dose groups (*p* = 0.005) (24). Yet, a PTV margin of at least 10 mm in cranio-caudal direction for breath-hold or gating approaches was specified in the protocol (24). As recalculation of delivered doses from CBCT used for image guidance was not reported in past randomized trials for stage III NSCLC, it cannot be ruled out that deviation of delivered from prescribed doses may be an important factor affecting outcome for individual patients.

In this study, D_min_ within the CTV was found to be the most important factor predicting *g*EUD_i_. The primary effectiveness measure in this study, D_min_ as a dosimetric measure, has been related to tumor recurrences in nasopharyngeal carcinoma (46). For lung cancer, D_min_ within the gross tumor can also be sensitive to the dose-calculation algorithm. Older, more simple algorithms as the type-C algorithm used in this study, which do not take into full account the lateral electron transport in heterogeneous media, tend to overestimate D_min_ in solid tumors surrounded by lung tissue (47, 48). As D_min_ was the dominant predictive feature and residual deformations at distinct points as HDP_worst_ and PD_min_global_ and the HDD_i_ do not allow precise prediction of the *g*EUD_i_, D_min_ should be considered during online image guidance. Without online adaptive replanning, the deformed CTV_i_ can be centered within the dose cloud in such a way that the encompassing isodose is maximized. A similar type of dose guidance was described by Smyth et al. for prostate cancer (49). The static dose cloud approximation (31) underlying this concept without dose recalculation was shown to hold sufficient accuracy in the present study. These results are in accordance with the findings of Valdes using Monte Carlo simulation (50).

Most parts of this workflow are implemented in the ETHOS™ adaptive radiotherapy platform or MR-linacs, allowing online adaptive replanning, however at the expense of considerably prolonged treatment times (51–53). On the contrary, dose guidance without dose re-optimization can be performed faster and more efficient. The present study showed that D_min_ is not well substituted by other parameters from the low-dose region of the dose–volume histogram for the CTV, as the correlation between D_min_ and D99.9, D99, D98, and D50 rapidly declined. Normalized D_min_ values >60% were associated with predicted *g*EUD_i_-values above 95% in the present study.

Systematic and random deviations of the distances between HDP_worst_ and D__PDmin_global_ points, the points on the CTV margins with the largest geometric and dosimetric deviations throughout the series in this study, were similar to displacements between the primary tumor and lymph nodes in the study by Weiss et al. (16). However, PTV margin calculation from these displacements according to the van Herk formula is not warranted, as the dose gradients around the PTV are rather heterogeneous for locally advanced NSCLC and the assumptions for dose accumulation in rigid CTVs are not completely fulfilled in the deformed targets in lung cancer (13).

A vital part for precise dose accumulation is deformable image registration (DIR). Respecting AAPM Task Group 132 guidelines for quality control of DIR algorithms, visual inspection of deformation results is indispensable. Our preliminary analysis showed that both deformation algorithms, SmartAdapt and MiM, resulted in important deviations from real anatomy in 14/218 fractions. Unsupervised uncertainty estimation of the deformation vector fields on the bases of differences between commercially available deformation algorithms alone as discussed by Amstutz et al. (54) remains investigational to our opinion.

In previous studies on dose accumulation, only minor deviations were found from intended goals in stage-III NSCLC. Wang et al. found a maximum decline in D95% for the PTV by 0.1% (range, −7.6%–5%) in 27 patients with stage-IIIA/IIIB NSCLC using five CBCTs per patient (55). The Aarhus (ART) group performed adaptive radiotherapy of patients with stage-III NSCLC with CBCT image guidance according to a soft tissue tumor match using a PTV margin of 4 mm around the primary tumor (56). Adaptive replanning throughout the course of series was applied if changes above an intervention level were found. The ART group compared the outcome with that from patients after bony matching using considerably larger PTV margins of 10 mm around the primary tumor without replanning. One from 52 in the ART group showed a marginal failure, while 4 from 52 in the no-ART group suffered from a marginal failure, supporting adaptive radiotherapy (56). The present study showed that the *g*EUD for the CTV from the accumulated dose distribution is close to the median of the distribution to the *g*EUD_i_s from the different dose fractions. Thus, deviations in a minority of *g*EUD_i_s were tolerated. This can be explained by the randomness of the residual deformations at the CTV margin, helping to avoid accumulation of dose minima at a single point so that the CTV minimum doses for the accumulated distributions stayed above 72%.

In the present study, all patients received induction chemotherapy and had a partial response. In the land mark trial Pacific (57, 58), 27% of all patients received induction chemotherapy. After partial response on induction chemotherapy, it is known that the tumor changes are minor during the course of radiochemotherapy (59). In this study, too, no substantial changes in the volume of the macroscopic tumor were observed that would have necessitated replanning.

A potential limitation of the results of the present study and a challenge for their translation into clinical workflow is that the deformations need to be monitored by an experienced radiologist and radiation oncologist for an accurate assessment of CTV coverage. To date, there is no (semi-)automated tool that could replace this important task. In addition, it is important to calculate *g*EUD_i_ and accumulated dose after supervised approval of CTV_i_s, each of which demands a thorough knowledge of the matter required. Randomness of the residual deformations of the CTV_i_ assured that D_min_ and *g*EUD of the accumulated dose distribution stayed above 70% and 100% of the prescribed dose, respectively. D_min_ proves to be a very important predictive feature for *g*EUD and outperforms geometric features associated with deformation. D_min_ was the most important parameter for *g*EUD prediction within the CTV for a single-dose fraction. Thus, the location and the value of D_min_ within the CTV are very important information for the evaluation of the CBCT during online image guidance.

## Conclusion

Residual deformations of the CTV after online image guidance affected the distribution of the *g*EUD_i_ per fraction but not the EUD_acc_ for the accumulated dose distribution over series of more than 10 fractions using conventional PTV margins of 5 mm in lung cancer. The smaller is the number of fractions, the more the individual *g*EUD_i_ will determine the outcome of the treatment series. D_min_i_ and not geometric parameters characterizing the residual deformation of the CTV was important for the prediction of the *g*EUD_i_. Dosimetric information during image guidance, at least as isodose contours linked to the planning CT, should be displayed, in order to center the CTV within the scheduled dose distribution to maximize the effectiveness of radiotherapy.

## Data availability statement

The original contributions presented in the study are included in the article/**Supplementary Material**. Further inquiries can be directed to the corresponding author.

## Ethics statement

The studies involving human participants were reviewed and approved by Ethics committee of University Duisburg-Essen. Written informed consent for participation was not required for this study in accordance with the national legislation and the institutional requirements.

## Author contributions

Conceptualization, NG and MS. Methodology, NG and MS. Software, MS. Validation, NG, MG, MS. Formal analysis, MS. Investigation, NG. Resources, NG. Data curation, MS. Writing—original draft preparation, NG. Writing—review and editing, NG, MG, CP, WL, FI, SQ, SL, AS, TR, MS. Visualization, MS. Supervision, MS. Project administration, MS. Funding acquisition, MS. All authors contributed to the article and approved the submitted version.

## Supplementary material

Supplementary Figure 1 new. Block diagram highlighting means and direction of 6 degrees of freedom (6DoF_i_) rigid online registration and deformation (DIR_i_) for obtaining the minimum dose D_min_i_ and the Hausdorff point HDP_i_ for the deformed clinical target volume CTV_i_ on the cone beam CT per fraction i. HDP_worst_ is the Hausdorff point with the maximum deformation over all HDPi, PD_min_global_i_ is the point with the minimum D_mini_ over all fractions. HDP_worst_ and PD_globalmin_ were identified on the respective CTV_i_, then back-deformed to CTV_plan_, and then deformed to the CTV_i_ for all other dose fractions using the respective deformations. Dashed line denoting deformable registration (DIR_i_), drawn line indicating the performed 6DoF_i_ rigid online registration per fraction i. DIR^-1^_i_ and 6DoF^-1^_i_ are the inverse deformations or rigid registrations.

Supplementary Figure 2 new. Block diagram delineating the process of deformable image registration (DIR) in addition to the 6DoF (6 degrees of freedom online match) for final dose accumulation. Stirred line denoting deformable registration. 6Dof^-1^_i_: the inverse of the 6 degrees of freedom online match vector for dose fraction i; DIR_i_: density based deformable image registration; CT_plan_: planning CT, CBCT_i_: pretreatment cone beam CT from fraction i; CTV_i_: clinical target volume contour for dose fraction i.
