# Supplementary figures and images for: Machine-learning-based prediction of the effectiveness of the delivered dose by exhale-gated radiotherapy for locally advanced lung cancer: The additional value of geometric over dosimetric parameters alone
Source: Front Oncol. 2023 Jan 13;12:870432. doi: 10.3389/fonc.2022.870432 (PMC9880443; doi:10.3389/fonc.2022.870432)

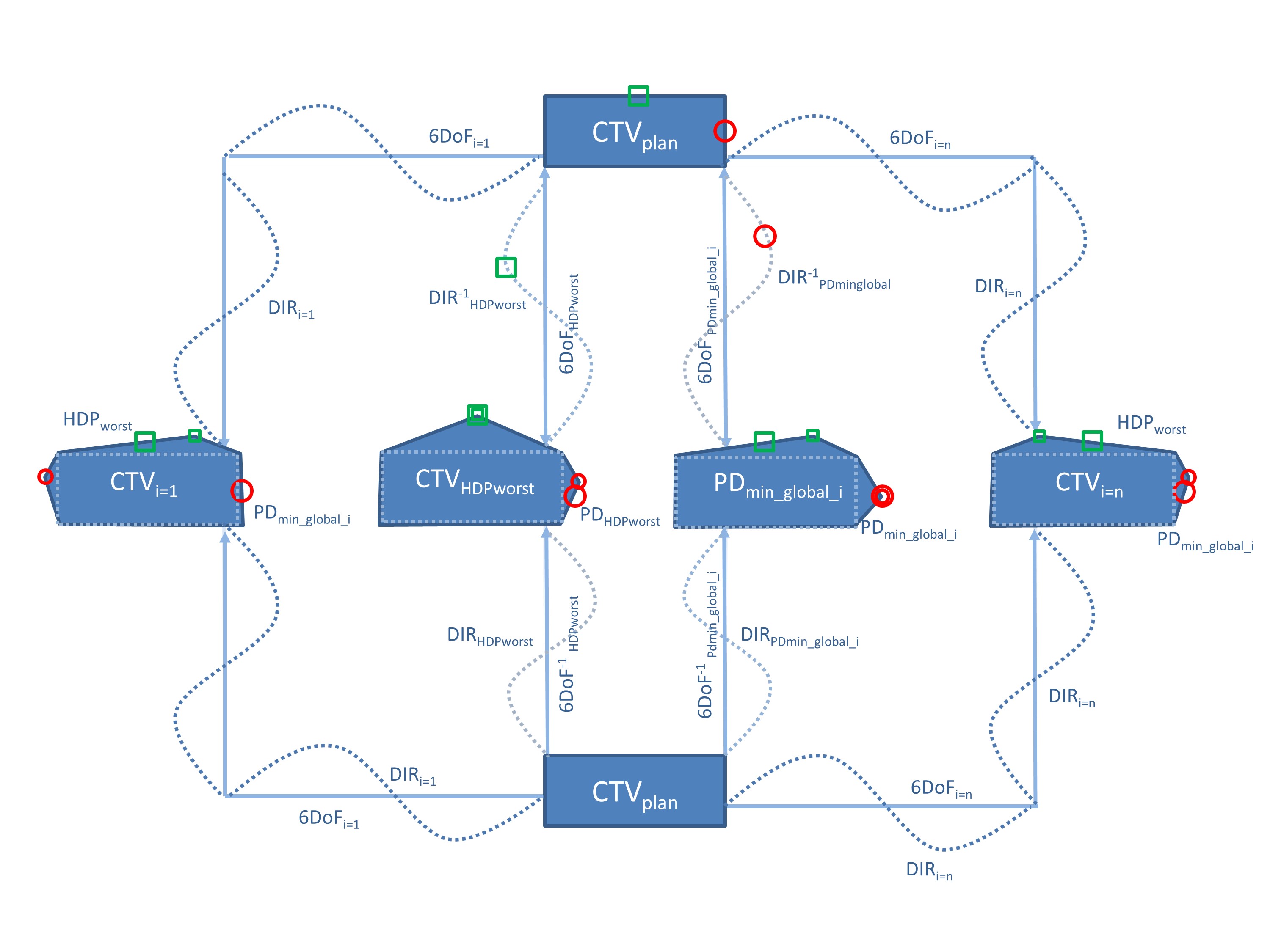

Supplement: Supplementary Figure 1 — new. Block diagram highlighting means and direction of 6 degrees of freedom (6DoFi) rigid online registration and deformation (DIRi) for obtaining the minimum dose Dmin_i and the Hausdorff point HDPi for the deformed clinical target volume CTVi on the cone beam CT per fraction i. HDPworst is the Hausdorff point with the maximum deformation over all HDPi, PDmin_global_i is the point with the minimum Dmini over all fractions. HDPworst and PDglobalmin were identified on the respective CTVi, then back-deformed to CTVplan, and then deformed to the CTVi for all other dose fractions using the respective deformations. Dashed line denoting deformable registration (DIRi), drawn line indicating the performed 6DoFi rigid online registration per fraction i. DIR-1 i and 6DoF-1 i are the inverse deformations or rigid registrations. [file Image_1.jpeg]

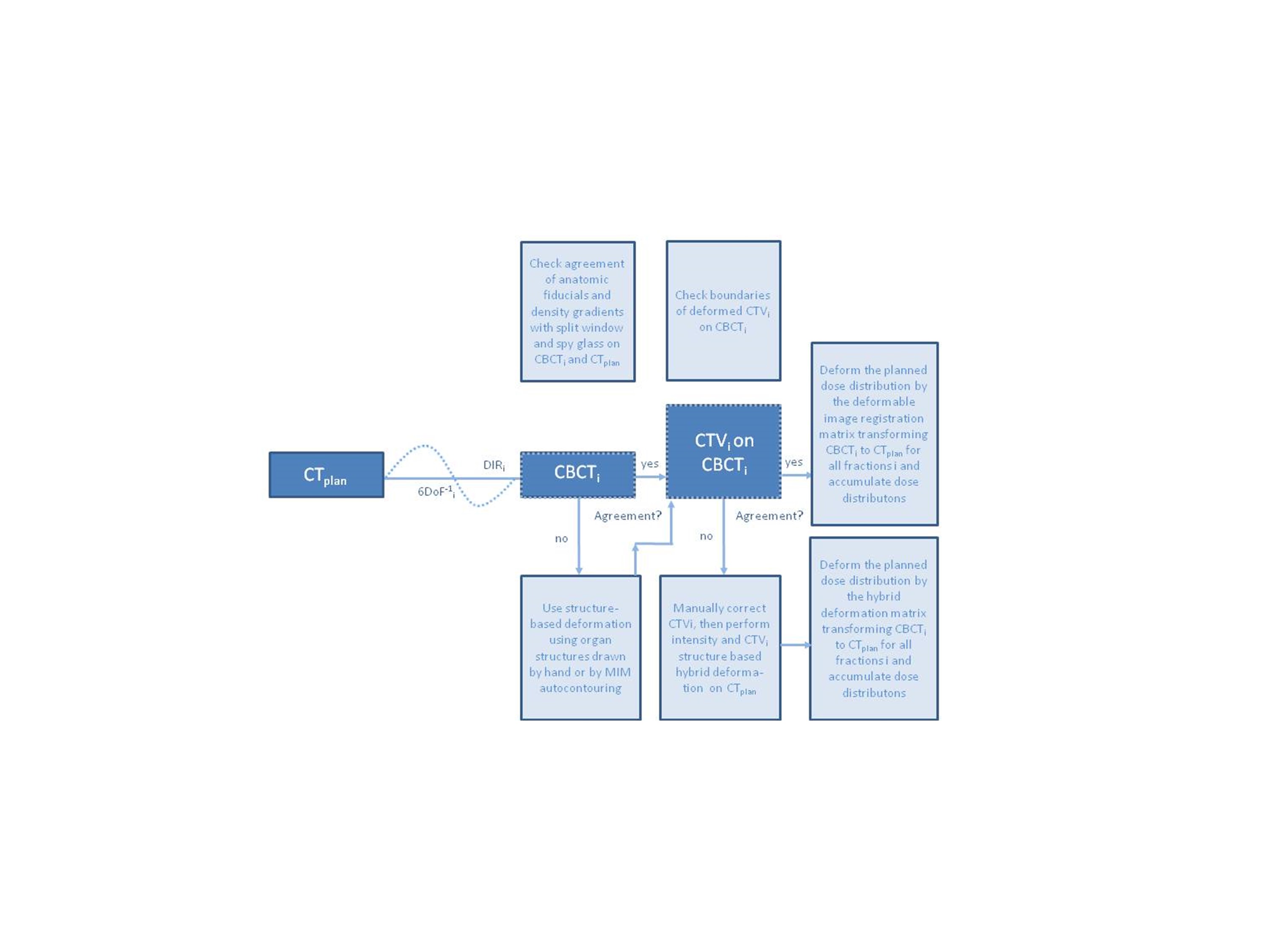

Supplement: Supplementary Figure 2 — new. Block diagram delineating the process of deformable image registration (DIR) in addition to the 6DoF (6 degrees of freedom online match) for final dose accumulation. Stirred line denoting deformable registration. 6Dof-1 i: the inverse of the 6 degrees of freedom online match vector for dose fraction i; DIRi: density based deformable image registration; CTplan: planning CT, CBCTi: pretreatment cone beam CT from fraction i; CTVi: clinical target volume contour for dose fraction i. [file Image_2.jpeg]
